# Statistical Modelling of Waning Immunity After Shanchol™ Vaccination: A Prospective Cohort Study

**DOI:** 10.3390/vaccines14020147

**Published:** 2026-01-30

**Authors:** Samuel Bosomprah, Fraser Liswaniso, Bernard Phiri, Mwelwa Chibuye, Charlie C. Luchen, Harriet Ng’ombe, Kennedy Chibesa, Dennis Ngosa, Mutinta Muchimba, Amanda K. Debes, Roma Chilengi, David A. Sack, Caroline C. Chisenga

**Affiliations:** 1 Basic Sciences and Immunology, Centre for Infectious Disease Research in Zambia, Lusaka 10101, Zambia; fraser.liswaniso@cidrz.org (F.L.); benard.phiri@cidrz.org (B.P.); mwelwa.chibuye@cidrz.org (M.C.); chaluma.luchen@cidrz.org (C.C.L.); harriet.ngombe@cidrz.org (H.N.); kennedy.chibesa@cidrz.org (K.C.); dennis.ngosa@cidrz.org (D.N.); mutinta.muchimba@cidrz.org (M.M.); chilengir@yahoo.com (R.C.); caroline.chisenga@cidrz.org (C.C.C.); 2 Department of Biostatistics, School of Public Health, University of Ghana, Accra P.O. Box LG13, Ghana; 3 Department of Epidemiology and Biostatistics, University of Zambia, Lusaka 10101, Zambia; 4 Department of Global Health, Amsterdam Institute for Global Health and Development, Amsterdam UMC, Location University of Amsterdam, 1105 AZ Amsterdam, The Netherlands; 5 Centre for Epidemic Response and Innovation, University of Stellenbosch, Stellenbosch 7600, South Africa; 6 Next Generation Sequencing and Division of Virology, Faculty of Health Sciences, University of the Free State, Bloemfontein 9300, South Africa; 7 Johns Hopkins Bloomberg School of Public Health, Johns Hopkins University, Baltimore, MD 21205, USA; adebes1@jhu.edu (A.K.D.); dsack1@jhu.edu (D.A.S.); 8 Zambia National Public Health Institute (ZNPHI), Lusaka 10101, Zambia; 9 Parasites and Microbes Programme, Wellcome Sanger Institute, Cambridge CB10 1SA, UK

**Keywords:** cholera, Shanchol™, vibriocidal, antibodies, waning

## Abstract

**Introduction**: Cholera remains a major public health threat in endemic settings, and oral cholera vaccine (Shanchol™) campaigns are increasingly used amid constrained global supply. However, practical decisions on revaccination require clearer, setting-specific estimates of how rapidly vaccine-induced vibriocidal antibodies peak and wane. **Methods**: We conducted a prospective cohort kinetics analysis in Lukanga Swamps (Central Province, Zambia), enrolling adults (18–65 years) stratified by prior Shanchol™ exposure (0, 1, or 2 previous doses). All participants received two Shanchol™ doses 14 days apart, with serum collected at baseline and days 14, 28, 60, and 90 (end of follow-up). Ogawa and Inaba vibriocidal titres were measured using a complement-based assay and analysed on the log10 scale. Serotype-specific mixed-effects models with natural cubic splines for time (knots: 14, 28, 60 days) assessed trajectories by prior-dose strata, adjusting for age, sex, and HIV status. Peak timing and post-peak half-life were derived from model-based predictions with participant-level bootstrap CIs (1000 replications). **Results**: The analysis included 225 participants: 68 (30.2%) with zero prior doses, 89 (39.6%) with one, and 68 (30.2%) with two; median age was 33 years (IQR 25–49), 56.4% were female, and 19.2% were HIV-positive. Modelled titres for both serotypes rose steeply after vaccination, peaking around day 36–37 across prior-dose strata. Ogawa titres reached half of peak by about day 73–78, corresponding to post-peak half-lives of 37–41 days; Inaba declined more slowly with half-lives of 42–46 days. Confidence intervals overlapped across prior-dose strata, indicating minimal differences by vaccination history. **Conclusions**: In this cholera-endemic adult population, Shanchol™ induced vibriocidal responses that peaked at ~5 weeks and waned over the following 5–7 weeks, with broadly similar kinetics regardless of prior vaccination and slightly slower decay for Inaba than Ogawa. These parameters can inform booster timing in hotspot settings.

## 1. Introduction

Cholera remains a serious public health threat in endemic regions, causing approximately 2.9 million cases and 95,000 deaths globally each year [1]. Sub-Saharan Africa bears a substantial burden, with frequent and often severe outbreaks reported over the past decades [2]. Zambia, for example, experienced a major cholera epidemic in 2017–2018 (~5900 cases), and the Lukanga Swamps area in Central Province, a known cholera hotspot, suffered a deadly outbreak in 2016 with a 7.4% case-fatality rate [3]. In response, oral cholera vaccines (OCVs) such as Shanchol™ have been deployed in these high-risk communities [4]. While reactive OCV campaigns have helped curb cholera in outbreak settings, the disease continues to resurge even in previously vaccinated populations [5]. This ongoing transmission suggests potential gaps in the durability of vaccine-induced immunity and/or coverage in vulnerable areas, underscoring the need for improved understanding of post-vaccination immunity in cholera-endemic regions.

In the face of persistent cholera endemicity in Africa and other regions, coupled with constrained vaccine resources, understanding the kinetics of immunity elicited by OCVs is crucial for guiding effective vaccination strategies worldwide. Although mass OCV campaigns have substantially reduced cholera incidence, vaccine-induced immunity wanes over time, and cholera can resurge in previously vaccinated communities; however, limited data on post-OCV immune kinetics make it difficult to predict when booster doses should be administered [6]. In fact, the ongoing global OCV shortage since 2022 has forced outbreak campaigns to adopt single-dose strategies [7] which provide significantly shorter protection (~1 year) than the standard two-dose regimen (~3 years) [8]. These circumstances underscore the importance of determining how rapidly OCV-induced antibodies rise and wane, to optimise the timing of re-vaccination campaigns [3,6]. Serum vibriocidal antibody titres, directed mainly against *Vibrio cholerae* O1 lipopolysaccharide, are the primary immunological correlate of protection [9], typically peaking within ~1–2 weeks after vaccination and then declining over subsequent months [6,9]. Therefore, evaluating the peak magnitude and decay rate (half-life) of vibriocidal responses under different conditions is critical. For example, prior OCV exposure can induce long-lived immunologic memory that yields faster or more durable antibody responses upon re-vaccination [10], and immune kinetics may also vary by age and sex (young children lose OCV-derived immunity sooner than adults, and females often mount stronger vaccine antibody responses than males) [11].

This study aimed to characterise the vibriocidal antibody kinetics over 90 days following oral cholera vaccination in a previously vaccinated cholera-endemic population. Comparing these antibody kinetics between individuals with different prior OCV exposure (e.g., zero, one, or two doses received in the past) can reveal whether previously vaccinated persons mount more rapid or durable responses upon boosting than OCV-naïve persons. Additionally, examining demographic factors is warranted, since age-related immune maturity or decline and sex-based immunological differences could potentially modulate vaccine responses.

## 2. Materials and Methods

### 2.1. Study Design and Participants

This kinetics analysis used a prospective cohort design conducted in Lukanga Swamps, a cholera hotspot in Central Province, Zambia, and was nested within the broader oral cholera vaccine (OCV) immunogenicity work previously described [6]. Participants were community members aged 18–65 years and were enrolled into prior Shanchol™ exposure strata (0, 1, or 2 previous doses); key exclusions included recent OCV receipt (within the past 12 months), current or recent diarrhoeal illness (within the past 2 weeks), recent antibiotic use (within the past 7 days), pregnancy, or participation in a similar vaccine study. All enrolled participants received two Shanchol™ (manufactured by Shantha Biotechnics Private Limited, India Shantha Biotechnics Limited, Hyderabad, India) doses administered 14 days apart and contributed repeated blood samples at baseline and at days 14, 28, 60, and 90 to characterise short-term serotype-specific vibriocidal antibody kinetics; further operational details are provided in the original paper [6].

### 2.2. Ethics Statement

Ethical approval for the parent study was obtained from the University of Zambia Biomedical Research Ethics Committee with the following reference number 007-12-16. Study authorisation was granted by the National Health Research Authority. All participants provided written informed consent, and all study procedures were conducted in accordance with Good Clinical and Laboratory Practices (GCLP).

### 2.3. Laboratory Procedures

Laboratory procedures have been described in detail elsewhere [6] and are summarised here for context. Serum vibriocidal antibody titres against *V. cholerae* O1 Ogawa (PIC158) and Inaba (PIC018) were measured at each study time point using a guinea pig complement-based assay. Briefly, colonies from overnight cultures were inoculated into Brain Heart Infusion broth, incubated at 37 °C for ~4 h, and harvested; heat-inactivated serum, exogenous guinea pig complement, and bacterial cells were combined in 96-well microtitre plates and incubated at 37 °C. Vibriocidal titres were defined as the reciprocal of the highest serum dilution producing a 50% reduction in optical density relative to positive control wells without serum (read at 595 nm), and seroconversion was defined as a ≥4-fold rise from baseline. In addition, anti-LPS plasma IgA, IgG, and IgM responses were quantified using a standardised ELISA: plates were coated with LPS (2.5 µg/mL), incubated with diluted plasma (1:50), probed with HRP-conjugated goat anti-human isotype-specific antibodies, developed using ABTS/H_2_O_2_, and read kinetically at 405 nm.

### 2.4. Statistical Analysis

Baseline characteristics of participants were summarised using frequency and proportion for categorical variables, while median and interquartile interval was used for continuous variables. Vibriocidal antibody kinetics against *V. cholerae* Ogawa and Inaba were modelled using repeated-measures mixed-effects models, complemented by model-based estimates of antibody half-life. Vibriocidal titres were analysed on the log10 scale because of their right-skewed distribution. Ogawa and Inaba titres were modelled separately. Time since first vaccination (in days) was entered flexibly using a natural cubic spline with internal knots at 14, 28, and 60 days, chosen a priori to capture the rapid rise and early decline in titres within the first three months. Age (15–25, 26–45, ≥46 years) and sex (male, female) were treated as categorical variables, as was HIV status (positive vs. negative). Prior exposure to Shanchol™ was coded as 0, 1, or 2 previous doses and is referred to as the “previous vaccination dosage”.

For each serotype, we fitted linear mixed-effects regression models of log10 titre on time, previous vaccination dosage, and their interaction, adjusting for age category, sex, and HIV status. Specifically, the fixed-effects component included main effects for previous vaccination dosage, the spline basis functions for time, and their interaction (previous doses × spline(time)), together with indicator variables for age category, sex, and HIV status. A random intercept for participant ID was included to account for within-person correlation of repeated measurements; we assumed an identity covariance structure at the participant level and an independent residual error. We also tested inclusion of a random slope for time, which improved the model fit and was retained. Models were fitted by maximum likelihood. All available titre measurements contributed under the missing-at-random assumption; no imputation of missing titres was performed. In sensitivity analyses, altering the placement of spline knots (by ±7 days) had a negligible impact on the estimated half-lives. Additionally, a bi-exponential decay model yielded similar half-life estimates for the initial waning phase and did not significantly improve the fit, supporting the use of the chosen spline model.

**Figure 1:**
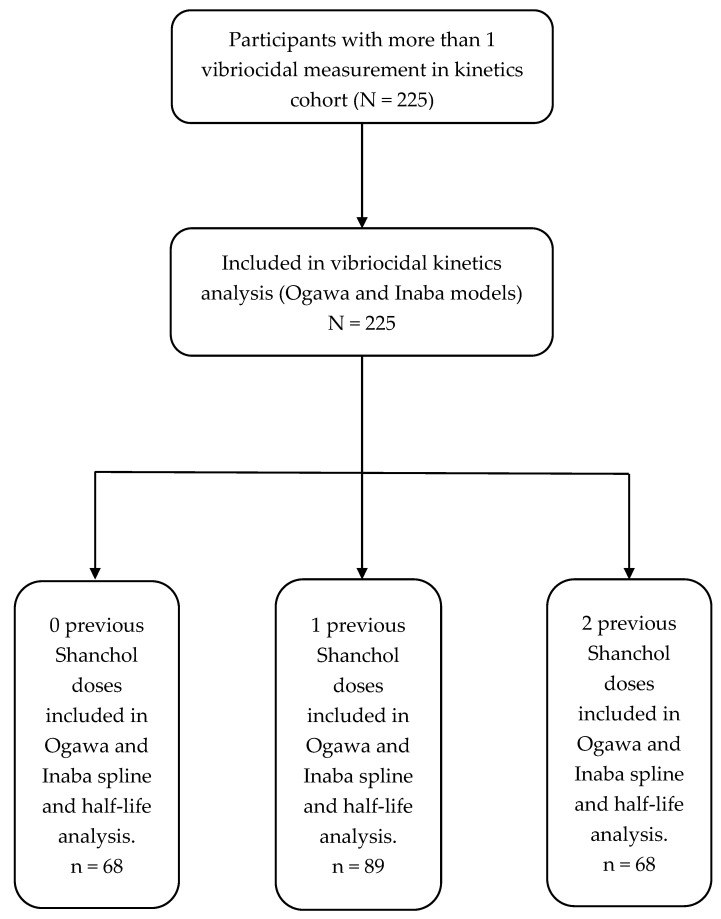
Participant flow.

**Figure 2:**
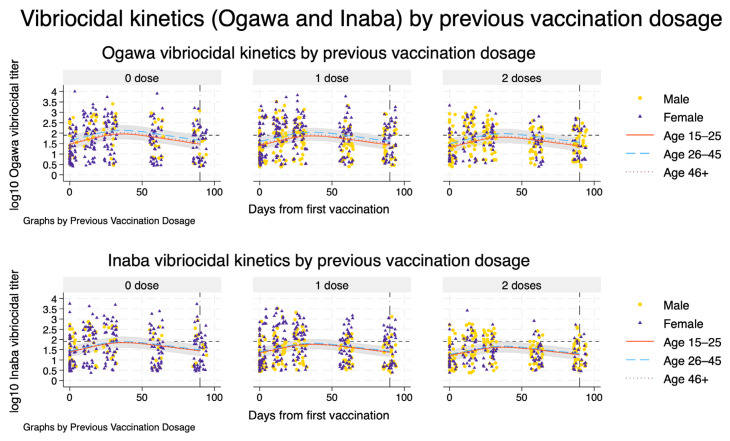
Serotype-specific vibriocidal kinetics by previous Shanchol™ vaccination history. Model-based trajectories of log_10_ vibriocidal titres to *V. cholerae* O1 Ogawa (top row) and Inaba (bottom row) over 90 days following the first vaccination, stratified by previous Shanchol™ doses (0, 1, or 2; columns). Within each panel, solid, dashed, and dotted lines represent predicted mean titres for adults aged 15–25, 26–45, and ≥46 years, respectively, for a reference male, HIV-negative participant. Shaded bands show the corresponding model-based 95% confidence intervals. Overlaid points show individual observed log_{10} titres, with circles and triangles indicating male and female participants, respectively. The horizontal dashed line marks the vibriocidal positivity threshold, and the vertical line at day 90 indicates the end of the primary follow-up window used for kinetic and half-life estimation.

From each fitted spline-based mixed model, we obtained model-based estimates of the population-average mean trajectory. To do this, we generated a prediction grid over time and covariate patterns. For Figure 1, we predicted log10 titres at daily intervals from day 0 to day 90 for each combination of previous vaccination dosage (0, 1, or 2 doses) and age category (15–25, 26–45, ≥46 years), holding sex fixed at male and HIV status at negative to represent a typical participant. Natural spline basis variables were recomputed on this grid, and we used the fitted models to obtain linear predictions (fixed-effects component only) and their standard errors. Pointwise 95% confidence intervals (CIs) for the mean log10 titre were derived using the standard normal approximation. For visualisation, these predicted mean trajectories and corresponding CIs were plotted alongside the observed log10 titres for each dose arm, stratified by age group, with sex-specific markers for the observed data. The same procedure was then repeated to extrapolate trajectories up to 365 days after vaccination (Figure 2), using a prediction grid from day 0 to day 365 with time modelled via the same spline basis.

To summarise the durability of the vibriocidal response in a clinically interpretable way, we used the fitted spline models to derive serotype- and dose-specific estimates of antibody “half-life” on the titre scale. For each serotype and previous vaccination dosage (0, 1, or 2 doses), we generated model-based predictions of the mean titre at daily intervals from day 0 to day 365 for a reference profile (male, 26–45 years, HIV-negative). Within this predicted trajectory, we first identified the peak titre as the maximum predicted value between day 0 and day 90 and recorded the earliest day at which this peak occurred (t_peak). We then defined the half-peak level as 50% of the peak titre and found the first subsequent day on which the predicted titre fell to or below this half-peak value (t_half). The model-based post-peak half-life was calculated as the difference, t_half–t_peak, expressed in days. If the predicted trajectory did not fall to half of its peak by day 365, the half-life for that dose arm was treated as undefined and left missing.

We estimated the uncertainty around the half-life using a non-parametric bootstrap at the participant level. For each serotype, we resampled participants with replacement (cluster bootstrap on participant ID) and refitted the spline-based mixed model in each bootstrap sample. For every bootstrap replication, we recomputed the serotype- and dose-specific peak day, half-peak day, and post-peak half-life using the algorithm described above. We generated 1000 bootstrap replications per serotype. For each quantity of interest (t_peak, t_half, and half-life) and for each dose arm, we took the median of the observed sample as the point estimate and the 2.5th and 97.5th percentiles of the bootstrap distribution as the 95% percentile confidence interval. All statistical tests were two-sided, and we considered a *p*-value < 0.05 and non-overlapping 95% confidence intervals as evidence of statistical significance, without formal adjustment for multiple comparisons, given the primarily descriptive and exploratory nature of the kinetic and half-life analyses. All statistical analyses were performed using Stata 19 (StataCorp, College Station, TX, USA) and the Stata do-file for the entire analysis workflow can be found in Supplementary File S1.

***Spline-based mixed model specification***

The model specification is as formulated below:

$$\begin{array}{c} y_{ij}=\beta_{0}+u_{i}+\beta_{T1}X_{i1}+\beta_{T2}X_{i2}+\sum_{k=1}^{K}\left[{(\beta }_{k}+\beta_{kT1}X_{i1}+\beta_{kT2}X_{i2})B_{k}\left(t_{ij}\right)\right]\\ +\beta_{A2}A_{i2}+\beta_{A3}A_{i3}+\beta_{F}F_{i}+\beta_{H}H_{i}+\varepsilon_{ij} \end{array}$$

where

$y_{ij}={log}_{10}\left({titer}_{ij}\right)$: log_10_-transformed vibriocidal titre for participant i at visit j.

$t_{ij}$: days since first vaccination (time variable “day” in the data).

$B_{k}\left(t_{ij}\right)$: k-th natural cubic spline basis function of time, evaluated at $t_{ij}$.

○ In practice, we used K = 2 spline basis functions (s_day1, s_day2) generated with mkspline, cubic knots (14 28 60) in Stata 19, corresponding to interior knots at 14, 28, and 60 days.

$X_{i1}$, $X_{i2}$: indicator variables for previous vaccination status

○ $X_{i1}$ = 1 if participant i had 1 previous Shanchol dose, 0 otherwise;
○ $X_{i2}$ = 1 if participant i had 2 previous Shanchol doses, 0 otherwise;
○ The reference group is participants with 0 previous Shanchol™ doses.

The terms $\beta_{k}B_{k}\left(t_{ij}\right)$ describe the average time course in the 0-dose group, and $\beta_{kT1}$ and $\beta_{kT2}$ allow the time trajectory to differ for the 1-dose and 2-dose groups (time x dose interaction).

Covariates:

$A_{i2}$, $A_{i3}$: age-group indicators

○ $A_{i2}$ = 1 for age 26–45 years, 0 otherwise;
○ $A_{i3}$ = 1 for age 46+ years, 0 otherwise;
○ The reference age group is 15–25 years.

$F_{i}$: biological sex indicator ($F_{i}$ = 1 for female, 0 for male).

$H_{i}$: HIV status indicator ($H_{i}$ = 1 for HIV-positive, 0 for HIV-negative).

Random effects and errors:

$u_{i}\sim N(0,\;{\delta_{u}}^{2})$: subject-specific random intercept, capturing between-participant variability in baseline log_10_ titre.

$\varepsilon_{ij}\sim N(0,\;\sigma^{2})$: residual error term, assumed independent of $u_{i}$.

All β parameters are fixed-effect regression coefficients to be estimated.

## 3. Results

### 3.1. Participant Flow and Background Characteristics of Participants

A total of 225 stored samples were included in the vibriocidal kinetics analysis for Ogawa and Inaba, distributed across prior Shanchol™ exposure as zero doses: 68 (30.2%), one dose: 89 (39.6%), and two doses: 68 (30.2%) (Figure 1). Overall, the median age was 33 years (IQI 25–49), with a clear age gradient by vaccination history (*p* < 0.001): median age increased from 26 [IQI 21–37] in the zero-dose group to 32 [IQI 25–49] in the one-dose group and 48 [IQI 31–55] in the two-dose group. Consistent with this, the age-category distribution differed strongly across dose strata (*p* < 0.001), with the zero-dose group having the largest share aged 15–25 years (45.5%), while the two-dose group was predominantly ≥46 years (53.8%) (Table 1). Sex also differed by prior vaccination status (*p* = 0.002). In the overall sample, 56.4% were female and 43.6% were male; however, the proportion of males increased stepwise from 27.9% (zero doses) to 44.9% (one dose) and 57.4% (two doses). HIV status varied across dose groups as well (*p* = 0.006): overall, 19.2% were HIV-positive, with the highest prevalence in the zero-dose group (31.3%), compared with 11.2% among those with one dose and 17.6% among those with two doses (Table 1).

### 3.2. Serotype-Specific Vibriocidal Kinetics by Previous Shanchol™ Vaccination History

Model-based trajectories of vibriocidal responses showed a rapid rise in both Ogawa and Inaba titres after the first Shanchol™ dose, with little visual distinction by previous vaccination history (Figure 2). Across all three prior-dose strata (zero, one, and two doses), titres increased steeply during the first month, reached their maximum around 5–6 weeks, and then declined gradually, and by day 90 had fallen close to the positivity threshold. The fitted curves for the three age groups (15–25, 26–45, and ≥46 years) were broadly similar in shape within each vaccination stratum, with only modest differences in peak height and overlapping 95% confidence bands, indicating limited age-related divergence in the overall kinetic pattern. Observed data points were scattered closely around the modelled trajectories, supporting a good visual fit for both serotypes and all arms.

Table 2 summarises the corresponding numerical estimates of peak timing and post-peak half-life for a reference participant (male, aged 26–45 years, HIV-negative). For Ogawa, modelled titres peaked at day 36 (95% CI: 35–38) in previously unvaccinated participants and at day 37 (95% CI: 35–40) in those with one or two prior Shanchol™ doses. The day at which titres fell to 50% of their peak (t_half) ranged from day 73 (95% CI: 67–82) in the zero-dose group to day 78 (95% CI: 69–96) in the two-dose group, corresponding to post-peak half-lives of 37–41 days with overlapping confidence intervals. For Inaba, peak timing was very similar (day 37 across all strata), but titres declined more slowly: t_half occurred between days 79 and 83, with post-peak half-lives of 42–46 days, again with overlapping 95% CIs across prior-dose categories. Results indicate very similar peak timing for Ogawa and Inaba responses, with Inaba showing slightly longer model-based post-peak half-lives and only small differences by previous vaccination history (Figure 2 and Table 2).

**Figure 3:**
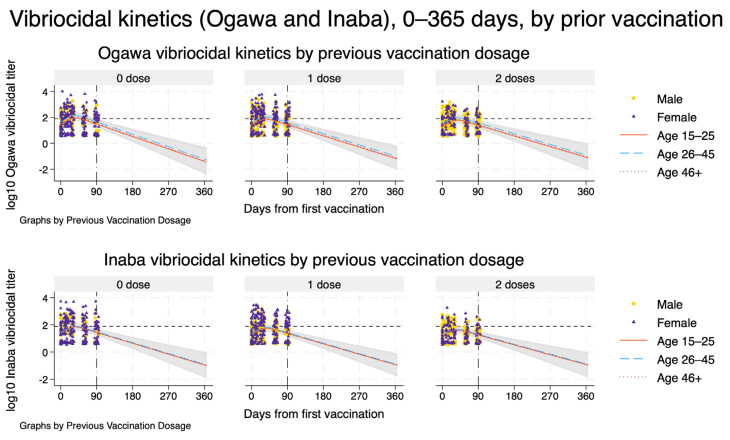
Model-based vibriocidal antibody kinetics against *V. cholerae* O1 Ogawa (**top row**) and Inaba (**bottom row**) from day 0 to day 365 after first vaccination, stratified by previous Shanchol™ dose. Each panel shows one prior vaccination stratum (0, 1, or 2 doses). Solid, dashed, and dotted curves represent model-predicted mean log_{10} vibriocidal titres for participants aged 15–25, 26–45, and ≥46 years, respectively, assuming male sex and HIV-negative status. Shaded ribbons indicate 95% confidence intervals around the predicted mean trajectories. Dots and triangles show observed log_{10} titres for men and women, respectively, within the empirical follow-up window. The vertical dashed–dot line at day 90 marks the end of the observed follow-up, beyond which curves represent model-based extrapolations only. The horizontal short-dashed line denotes the prespecified vibriocidal positivity threshold (titre 80).

Extending the spline mixed-effects models to 12 months after first vaccination showed that, for both Ogawa and Inaba, mean vibriocidal titres peaked around 5–6 weeks and then declined approximately log-linearly over time across all prior vaccination strata (Figure 3). The projected decay patterns were similar by previous Shanchol™ dose, with only modestly higher predicted titres among participants with two prior doses compared with those with none or one prior dose. By about three months, model-based mean titres were close to, or just above, the vibriocidal positivity threshold, and by 6–12 months the trajectories were projected to fall clearly below this threshold, with increasingly wide 95% confidence bands reflecting greater extrapolation uncertainty beyond the observed 90-day follow-up. Extrapolation beyond the last observed time point is therefore entirely model-based and should be interpreted with appropriate caution.

## 4. Discussion

In this cohort of adults from Lukanga Swamps, an outbreak-prone setting with heterogeneous prior exposure to Shanchol™, we observed broadly consistent serotype-specific vibriocidal antibody kinetics after re-vaccination, characterised by a clear rise in titres followed by gradual waning. Across prior-dose strata (zero, one, or two previous doses), the spline-based mixed-effects models suggested that both Ogawa and Inaba titres peaked around five weeks after the first dose (approximately day 36–37), after which titres declined over subsequent months. The estimated post-peak half-life was on the order of 5–6 weeks, with a tendency toward slower decay for Inaba (≈42–46 days) compared with Ogawa (≈37–41 days), though confidence intervals were wide and overlapped across strata. Notably, Inaba titres waned more slowly than Ogawa titres. While this serotype difference was modest and has been observed in other studies, it likely represents an immunologic phenomenon without a clear impact on protection or outbreak dynamics. Importantly, the dose strata also differed in baseline characteristics, participants with two prior doses were older and more often male, and HIV positivity was more frequent in the zero-dose stratum, highlighting the need to interpret apparent kinetic differences in light of underlying demographic and clinical heterogeneity. Taken together, these findings support the notion that, in this population, boosting elicits measurable serotype-specific responses that wane over a timeframe of weeks to months, providing quantitative parameters that can inform how rapidly antibody-mediated protection may diminish following vaccination in similar outbreak settings.

Waning of vibriocidal antibodies occurs relatively quickly after the peak, especially with killed OCVs in field settings. In our study, we noted a substantial titre decline within months of vaccination, which mirrors patterns seen in other endemic settings [12]. Researchers have cautioned that the single-dose OCV strategy may only afford brief protection, noting that a one-dose campaign’s effect can diminish after a few months [6]. Encouragingly, our data suggest that vibriocidal antibodies do not disappear entirely in the short term; titres at day 90, though reduced, were still elevated above baseline in all groups. Moreover, a study of a live-attenuated OCV (CVD 103-HgR) in immunologically naïve adults demonstrated remarkably durable titres, showing no significant decline from 3 to 12 months post-vaccination and an estimated vibriocidal half-life of ~860 days (over 2 years) [13]. This live-vaccine study predicted titres persisting at protective levels for at least two years. While killed OCVs in endemic settings likely have shorter-lived antibody responses than this ideal scenario, the live OCV data highlight that under optimal conditions, vibriocidal antibodies can be maintained long term. In summary, the consensus across studies is that vibriocidal titres peak early and then wane substantially within 3–6 months, especially in young children, although a fraction of the response may persist above baseline for 1–2 years. Our current findings on antibody decay align with this pattern of rapid initial waning, reinforcing concerns about the durability of OCV-induced immunity in high-risk settings. We emphasise that our study measured the kinetics of the vibriocidal antibody response over 90 days and did not directly assess immunologic memory or protection. While the waning of antibodies can inform booster scheduling, it does not equate to an absence of immunologic memory or a direct measure of clinical protection.

Prior immunologic exposure to cholera (through past vaccination or infection) can shape the kinetics and magnitude of the antibody response. Re-vaccination studies offer mixed evidence on the booster effect. In our study, adults re-vaccinated ~4 years after a previous two-dose regimen showed no significant advantage in antibody rise compared to OCV-naïve individuals. The overall vibriocidal kinetics were similar among all treatment arms regardless of zero, one, or two prior doses in our study, indicating that memory from 4 years prior did not markedly heighten the early antibody peak or alter the decay rate. However, this similarity in kinetics across previously vaccinated and unvaccinated adults may reflect a ceiling in vibriocidal antibody levels or high baseline immunity from endemic exposure, rather than an absence of immunologic memory. In contrast, longer-term priming in young children may yield a more pronounced booster effect. Chowdhury et al. (2020) [14] in Bangladesh examined children who had received a single OCV dose more than 3 years earlier and then received a booster dose. They observed augmented immune responses in the primed group: vibriocidal titres two weeks after the booster were significantly higher in previously vaccinated children < 5 years old than in age-matched first-time vaccinees [14]. Young children with prior OCV had a brisk anamnestic response (including elevated anti-LPS IgA) upon boosting, whereas OCV-naïve children’s responses were more modest [14]. This suggests that a single priming dose can induce lasting immunological memory in children, which dramatically amplifies the response to a later dose. Notably, this booster was given ~3.3 years after the initial dose, slightly shorter than the 4-year gap in our study, and involved a paediatric population (who tend to have lower primary responses). These differences likely explain the divergent outcomes: in children, a booster after a few years elicited a clear rise above primary responses [14], whereas in adults, prior vaccination (especially if long ago) offered little immediate boost [6].

It is also important to consider natural exposure. Populations in endemic areas often have subclinical exposure to *Vibrio cholerae* that can act as a primer. For instance, the immunogenicity of OCV in Haiti (a historically cholera-naïve population) was lower after one dose compared to Bangladesh (endemic), but after two doses the gap closed [15]. This implies that people in Bangladesh had pre-existing immunity (from environmental exposure or past mild infections) that effectively made the first dose a “booster,” yielding higher titres. Likewise, a study of refugees from Myanmar found that the displaced population’s baseline vibriocidal titres were not significantly different from local Bangladeshis, suggesting prior exposure to cholera; correspondingly, their vaccine responses were comparable to endemic populations despite coming from a previously non-vaccinated group [14]. In our study area, which experiences periodic outbreaks, many participants likely had some immunological memory from past infections or OCV campaigns. Such background exposure may have mitigated differences between first-time and repeat vaccinees, as was seen in analyses from South Sudan where individuals with evidence of recent cholera exposure had higher odds of seroconversion, but overall, those with a prior OCV a year earlier responded just as well to vaccination as those without prior OCV (Iyer et al., 2016) [16]. In summary, the influence of prior vaccination on vibriocidal kinetics is context dependent. Prior doses can induce memory that boosts responses on re-vaccination, a phenomenon most evident in young children and shorter intervals, but in adults or with long gaps, a repeat course may behave immunologically much like a primary series.

A key strength of this study is the longitudinal design with repeated vibriocidal measurements at clearly defined post-vaccination time points (baseline, days 14, 28, 60, and 90), which allowed the team to characterise serotype-specific kinetics and to summarise clinically interpretable parameters (timing of peak response and post-peak half-life) rather than relying only on single time point contrasts. The use of mixed-effects spline models appropriately accounted for within-participant correlation, accommodated non-linear antibody trajectories, and enabled smooth prediction curves with uncertainty bands; importantly, the half-life summaries were derived from the fitted trajectories and accompanied by cluster bootstrap confidence intervals, providing a transparent quantification of uncertainty. Our half-life estimates pertain only to the initial, early decay phase. Stratification by prior Shanchol™ exposure and consideration of age and sex also enhanced relevance for programme decisions in settings where revaccination and heterogeneous exposure histories are common. Limitations should be noted. Follow-up ended at day 90, so projections beyond this window (e.g., to one year) rely on model extrapolation and should be interpreted cautiously, particularly if longer-term decay deviates from the early post-peak pattern (e.g., if a slower decay phase occurs beyond 90 days). The analysis was based on a specific cohort from a prior hotspot with substantial baseline heterogeneity (including differences in age, sex, and HIV status across dose strata), which may introduce residual confounding and may limit generalisability. Additionally, we did not observe a significant difference in antibody kinetics between HIV-positive and HIV-negative participants (no significant time × HIV interaction), although our study was not powered for a definitive subgroup analysis. In addition, vibriocidal titres, while the most widely used serologic correlate of protection, are an imperfect proxy for clinical protection and do not fully capture mucosal immunity or memory responses; thus, kinetic differences should be interpreted as immunologic signals rather than direct estimates of protection. Another important limitation is that our model and analyses considered only reported symptomatic cholera cases. In cholera epidemics, however, asymptomatic carriers (i.e., individuals infected with *V. cholerae* who do not develop symptoms) can make up a large fraction of all infections and yet continue to shed the bacteria, thereby sustaining transmission [17,18,19,20]. This means that the true infection count is higher than reported cases, and interventions or predictions based only on symptomatic cases may underestimate the epidemic potential. Again, relying solely on symptomatic case data may limit generalisability to the entire infected population.

These findings have practical implications for cholera preparedness and vaccination strategy because they translate short-term post-OCV immune responses into programmatically interpretable metrics, when titres peak and how quickly they decline, stratified by prior vaccination exposure and by serotype. In Zambia, where outbreaks can recur in known hotspots and vaccine supply constraints may necessitate flexible dosing strategies, the observed kinetics support using post-vaccination monitoring and trajectory-based summaries (rather than single time point comparisons) to inform the timing of booster campaigns, particularly in communities with mixed prior OCV exposure. The serotype-specific patterns also underscore the value of reporting Ogawa and Inaba responses separately, since aggregate measures may obscure meaningful differences relevant to local transmission dynamics. More broadly, for cholera-endemic and outbreak-prone settings facing periodic reactive campaigns and intermittent vaccine shortages, incorporating estimates of peak and post-peak half-life into decision-making can help refine booster intervals, prioritise high-risk subgroups, and improve the alignment between expected duration of immunologic protection and the seasonal or cyclical nature of outbreak risk, while highlighting the need for longer follow-up data to validate projected persistence beyond the observed window.

## 5. Conclusions

Using mixed-effects spline models, we showed that vibriocidal antibody responses to Shanchol™ rise to a clear post-vaccination peak within the first 5–6 weeks and then decline measurably over subsequent weeks, with broadly comparable kinetics across prior OCV dose strata but with modest evidence of more sustained post-peak persistence among those with greater prior exposure, and with Inaba responses tending to wane more slowly than Ogawa in the model-based projections. In Zambia, immunisation programmes could consider proactive planning for revaccination/boosting in known hotspots before periods of heightened risk, while recognising that protection may attenuate over months and that one-dose strategies used during supply constraints may require earlier boosting than standard two-dose approaches. We recommend that future work in similar settings extends follow-up beyond 90 days (ideally to ≥12 months), evaluates whether kinetic parameters differ by age and sex after accounting for baseline differences in prior exposure, and triangulates vibriocidal decay with additional correlates of protection (including mucosal and cellular markers) to strengthen inference about clinical relevance.

## Figures and Tables

**Table 1:** Background characteristics of participants.

Characteristics	Previous Shanchol™ Dose				
	0 Dose	1 Dose	2 Doses	Total	Test
N	68 (30.2%)	89 (39.6%)	68 (30.2%)	225 (100.0%)	
Age; median (IQI)	26 [21; 37]	32 [25; 49]	48 [31; 55]	33 [25; 49]	<0.001
Age (years)					
15–25	30 (45.5)	27 (31.4)	10 (15.4)	67 (30.9)	<0.001
26–45	25 (37.9)	34 (39.5)	20 (30.8)	79 (36.4)	
46+	11 (16.7)	25 (29.1)	35 (53.8)	71 (32.7)	
Sex					
Male	19 (27.9)	40 (44.9)	39 (57.4)	98 (43.6)	0.002
Female	49 (72.1)	49 (55.1)	29 (42.6)	127 (56.4)	
HIV status					
Negative	46 (68.7)	79 (88.8)	56 (82.4)	181 (80.8)	0.006
Positive	21 (31.3)	10 (11.2)	12 (17.6)	43 (19.2)	

Median (Interquartile Interval): *p*-value from Pearson’s chi2 test; Frequency (Percent%): *p*-value from Pearson test.

**Table 2:** Model-based estimates of peak timing and post-peak half-life of vibriocidal titres, by serotype and previous Shanchol™ vaccination history *.

Previous Shanchol™ Doses	Ogawa			Inaba		
	Day of Peak Titre (t_Peak), Median (95%CI)	Day Titre Reaches 50% of Peak (t_Half), Median (95%CI)	Post-Peak Half-Life (Days), Median (95%CI) †	Day of Peak Titre (t_Peak), Median (95%CI)	Day Titre Reaches 50% of Peak (t_Half), Median (95%CI)	Post-Peak Half-Life (Days), Median (95%CI) †
0 doses	36 (35–38)	73 (67–82)	37 (31–45)	37 (35–39)	79 (70–97)	42 (33–59)
1 dose	37 (35–38)	76 (69–87)	39 (33–50)	37 (35–39)	80 (71–95)	43 (36–57)
2 doses	37 (36–40)	78 (69–96)	41 (33–57)	37 (35–40)	83 (71–108)	46 (35–69)

* Estimates obtained from natural cubic spline mixed-effects models (knots at days 14, 28, and 60), evaluated for a reference participant (male, aged 26–45 years, HIV-negative); † Defined as the difference between t_half and t_peak (calendar days since first vaccination at which the model-based titre falls to 50% of its peak value). CIs of 95% were estimated using bootstrap with 1000 replications.

## Data Availability

All data generated and analysed during this study are included in the published manuscript and Supplementary Information File. The data presented in this study are available upon reasonable request from the corresponding author. The CIDRZ Ethics and Compliance Committee is responsible for approving such request. To request data access, one must write to the Secretary to the Committee/Head of Research Operations, Hope Chinganya (hope.chinganya@cidrz.org). Dataset requests must include contact information, a research project title, a description of the proposed analysis, and the format in which it is expected. The requested data should only be used for the purposes related to the original research or study. The CIDRZ Ethics and Compliance Committee will normally review all data requests within 48–72 h (Monday–Friday) and provide notification if access has been granted or additional project information is needed before access can be granted.

## Supplementary Materials

The following supporting information can be downloaded at https://www.mdpi.com/article/10.3390/vaccines14020147/s1, File S1: Stata do-file for the entire analysis workflow.
