# Supplementary material for: Statistical Modelling of Waning Immunity After Shanchol™ Vaccination: A Prospective Cohort Study
Source: Vaccines (Basel). 2026 Jan 30;14(2):147. doi: 10.3390/vaccines14020147 (PMC12945247; doi:10.3390/vaccines14020147)
Supplement: Supplementary file 1 [file vaccines-14-00147-s001.zip › vaccines-4111076-supplementary.pdf]

```
clear
set more off

cd "/Users/samuelbosomprah/Documents/Sam/Stuffs/CIDRZ/Dr CCC Chisenga/MS/
OCV Biomarkers /dataset"

use COMPILED_MEEDAT_Combined,clear

gen id_1=_n
order id_1

ren ogawatiter_0 ogawatiter0
ren ogawad14 ogawatiter14
ren ogawa_titerd30 ogawatiter28
ren ogawa_titerd60 ogawatiter60
ren ogawa_titerd90 ogawatiter90

ren inabatiter_0 inabatiter0
ren inabad14 inabatiter14
ren inaba_titer28 inabatiter28
ren inaba_titer60 inabatiter60
ren inaba_titerd90 inabatiter90

label var treatment_arm "Previous Vaccination Dosage"

sort id_1

keep if cohort==0 // for the decay model where titers are measured longitudinally

save COMPILED_MEEDAT_Combined_v1,replace

use COMPILED_MEEDAT_Combined_v1,clear

***
drop ogawatiter0 ogawatiter14 ogawatiter28 ogawatiter60 ogawatiter90 inabatiter0
inabatiter14 inabatiter28 inabatiter60 inabatiter90 seroconversion_ogawa
seroconversion_inaba // i need only the charactetics dataset to merge later after
reshape

sort id_1
save COMPILED_MEEDAT_Combined_covariates,replace

use COMPILED_MEEDAT_Combined_covariates,clear

label var age "Age; median(IQI)"
```

```
label var age_cat "Age (years)"
label var new_sex "Sex"
label var new_hiv_status "HIV status"
label var treatment_arm "Previous Shanchol dose "

tab1 age_cat new_sex new_hiv_status treatment_arm


dtable age i.age_cat i.new_sex i.new_hiv_status, ///
	by(treatment_arm, totals tests) ///
	define(iqi = q1 q3, delimiter("; ")) ///
	continuous(age, statistic(q2 iqi)) ///
	factor(,statistic(fvfrequency fvpercent)) ///
	nformat(%5.0f q2 q1 q3) ///
	sformat("[%s]" iqi) ///
	sformat("(%s)" fvpercent) ///
	column(summary(Number of participants (% of total))) ///
	title(Table 1: Background characteristics of participants) ///
	note(Median (Interquartile Interval): p-value from Pearson's chi2 test.) ///
	note(Frequency (Percent%): p-value from Pearson test.) ///
	export(table1.xlsx,replace)



*************** creating the longitudianl dataset
***************
use COMPILED_MEEDAT_Combined_v1,clear

order id_1 ogawatiter0 ogawatiter14 ogawatiter28 ogawatiter60 ogawatiter90
inabatiter0 inabatiter14 inabatiter28 inabatiter60 inabatiter90 seroconversion_ogawa
seroconversion_inaba

keep id_1 ogawatiter0 ogawatiter14 ogawatiter28 ogawatiter60 ogawatiter90 inabatiter0
inabatiter14 inabatiter28 inabatiter60 inabatiter90

reshape long ogawatiter inabatiter, i(id_1) j(day)

sort id_1

save kinetics,replace

use COMPILED_MEEDAT_Combined_covariates,clear

merge 1:m id_1 using kinetics
tab _merge
drop _merge
```

```
order id_1 day ogawatiter inabatiter
sort id_1 day

save kinetics_v1,replace




*------------------------------------------------------------*
* 0. PREP: Declare panel structure and check time variable    *
*------------------------------------------------------------*
use kinetics_v1,clear // load the dataset

* id = participant ID
* day = time in days post-vaccination/infection (0,14,28,60,90)
* titer = vibriocidal titre (continuous, positive)

xtset id_1 day    // declare panel data: each id measured at multiple days

summarize day ogawatiter   // quick check on ranges and missingness
tabulate day        // confirm you have 0, 14, 28, 60, 90


*------------------------------------------------------------*
* 1. LOG-TRANSFORM TITERS (handle zeros if present)          *
*------------------------------------------------------------*

* If you have no zero titers:
generate ln_ogawatiter = ln(ogawatiter)

* If some titers are recorded as 0 or below LOD, consider:
* - replacing 0 with 1/2 LOD or similar.
* Example (assuming LOD is known and stored as scalar lod):
* replace ogawatiter = lod/2 if ogawatiter == 0
* generate ln_ogawatiter = ln(ogawatiter)

summarize ogawatiter ln_ogawatiter
histogram ln_ogawatiter, normal   // sanity check: often closer to normal than raw titers


*------------------------------------------------------------*
* 2. LOG-LINEAR MIXED MODEL (EXPONENTIAL DECAY)              *
*------------------------------------------------------------*
/*
This is your primary "exponential decay" model:

 log(titer) = beta_0 + beta_1*{day} + u_{0i} + u_{1i}*{day} + epsilon_{it}
```

```
*/

* Random intercept and random slope for day, unstructured covariance
mixed ln_ogawatiter c.day || id: c.day, covariance(unstructured)

* View estimates
estimates store M_lin // Exponential decay model
estat ic            // get AIC/BIC for model comparison later


*-------------------------------------------------------------*
* 2a. Compute population-average half-life from fixed slope    *
*-------------------------------------------------------------*
/*
If the model is lntiter = b0 + b1*day, then titers decay as
T(t) = e^{b0}*exp(b1 t).
If b1 is negative (i.e., decay), the half-life is:

T_{1/2} = ln(0.5)}/{b_1} (i.e., the time it takes for the titer to decay to half of its initial
amount)

*/

* Check coefficient names if you want (optional)
ereturn list
matrix list e(b)

* Extract fixed-effect coefficient for day
scalar b_day = _b[day]
display "Fixed-effect slope (log-titer vs day) = " %9.4f b_day

* Only compute half-life if the slope is negative (decay)
capture drop t_half
if (b_day < 0) {
   scalar t_half = ln(0.5) / b_day
   display "Estimated half-life (days) = " %9.3f t_half
}
else {
   display as error "Note: Slope is >= 0 (b_day = " %6.4f b_day "), half-life not
meaningful."
}


*-------------------------------------------------------------*
* 2b. Plot: observed log-titers + population-average log curve *
```

```
*-----------------------------------------------------------*

* Predicted population-averaged log-titers (fixed effects only)
predict lntiter_hat_fe, xb   // fixed-only prediction on log scale

* Example plot: individual trajectories + population curve

twoway ///
   (scatter ln_ogawatiter day, connect(line) mcolor(gs12)) ///
   (line lntiter_hat_fe day, sort lwidth(medthick)), ///
   ytitle("Vibriocidal titer (log scale)") ///
   xtitle("Days post-vaccination/infection") ///
         legend(order(1 "Individual log-titers" 2 "Population-average (log-linear model)")
///
   position(12) ring(0) col(1) region(lstyle(none)))


*-----------------------------------------------------------*
* 3. QUADRATIC (POLYNOMIAL) MIXED MODEL                *
*-----------------------------------------------------------*
*log(titer) = beta_0 + beta_1*{day}+ beta_1*{day}^2+ ...

* Create polynomial term
generate day2 = day^2

* Mixed model with random intercept + random slope for day
mixed ln_ogawatiter c.day c.day2 || id: c.day, covariance(unstructured)

estimates store M_quad //QUADRATIC (POLYNOMIAL) MIXED MODEL
estat ic     // compare AIC/BIC to M_lin

* If you suspect curvature but want parsimony, you can:
*  - test whether day2 significantly improves fit:
test day2


* Likelihood-ratio test (M_lin nested in M_quad)
lrtest M_lin M_quad



*-----------------------------------------------------------*
* 4. SPLINE-BASED MIXED MODEL                       *
*-----------------------------------------------------------*

* Create cubic B-splines for day with knots at 14, 28, 60
* This will generate a set of spline variables: s_day1 s_day2 ...
```

```
mkspline s_day = day, cubic knots(14 28 60)

* Check what was created
describe s_day*


* Mixed model with spline basis as fixed effects
* Random intercept (and optionally random slopes on one spline component)
mixed ln_ogawatiter s_day* || id:, covariance(identity)

estimates store M_spline //Cubic B-splines, random intercept
estat ic        // AIC/BIC for comparison

* Add a simple random slope on day plus spline fixed effects
mixed ln_ogawatiter c.day s_day* || id: c.day, covariance(unstructured)

estimates store M_spline2 //Cubic B-splines, random intercept and slope
estat ic


*------------------------------------------------------------*
* 5. MODEL COMPARISON: AIC/BIC & LR tests                  *
*------------------------------------------------------------*

* Show ICs side by side
estimates stats M_lin M_quad M_spline M_spline2, all //M_spline2 is best (lowest BIC
and AIC)



*------------------------------------------------------------*
/* 6a. Numerical half-life from spline model: this is exactly
where the spline model differs from the simple exponential:
there's no closed-form half-life, so we have to derive it
numerically from the fitted curve.

Definition: Time from the peak predicted titer to the time when
        the predicted titer falls to 50% of that peak.*/
*------------------------------------------------------------*

use kinetics_v1,clear


* If you have no zero titers:
generate ln_ogawatiter = ln(ogawatiter)

* If some titers are recorded as 0 or below LOD, consider:
```

```
* - replacing 0 with 1/2 LOD or similar.
* Example (assuming LOD is known and stored as scalar lod):
* replace ogawatiter = lod/2 if ogawatiter == 0
* generate ln_ogawatiter = ln(ogawatiter)

summarize ogawatiter ln_ogawatiter
histogram ln_ogawatiter, normal   // sanity check: often closer to normal than raw titers


* Create cubic B-splines for day with knots at 14, 28, 60
* This will generate a set of spline variables: s_day1 s_day2 ...
mkspline s_day = day, cubic knots(14 28 60)

* Check what was created
describe s_day*

* Add a simple random slope on day plus spline fixed effects
mixed ln_ogawatiter c.day s_day* || id: c.day, covariance(unstructured)


//Step 1. Create a fine time grid (0–90 days)

* Create a fine time grid for prediction (0 to 90 days)
preserve
clear
set obs 91
generate day = _n - 1   // 0,1,2,...,90

//Step 2. Re-create the spline basis on this grid

* Re-create the spline basis for the new dataset
mkspline s_day = day, cubic knots(14 28 60)
describe day s_day* //You should see s_day1, s_day2 (as before).

//Step 3. Predict the population-average log-titers and titers

* Predict population-average log-titers (fixed effects only)
predict lntiter_hat_spline, xb
generate titer_hat_spline = exp(lntiter_hat_spline) //Convert to predicted titers on
original scale. Now titer_hat_spline is your smooth population curve from 0–90 days.

//Step 4. Find the peak predicted titer and its timing

* Find the peak predicted titer (0–90 days)
summarize titer_hat_spline, meanonly
scalar peak = r(max) //peak  = highest predicted titer
```

```
display "Peak predicted titer = " %9.3f peak

* Identify the earliest day at which the peak occurs
generate peak_flag = (titer_hat_spline == peak)

summarize day if peak_flag, meanonly
scalar t_peak = r(min) //t_peak = day at which the predicted titer reaches its maximum
(often around day ~40–50 for your quadratic/spline shape)

display "Peak day (earliest) = " %9.3f t_peak


//Step 5. Define the half-peak level

* Define half of the peak as the target level
scalar half_peak = peak/2
display "Half-peak titer = " %9.3f half_peak


//Step 6a. Find the half-life as "time to 50% of peak after the peak"

* Mark days at or after the peak
generate after_peak = (day >= t_peak)

* Mark days where titer has dropped to or below half-peak AND we are after the peak
generate below_half = (titer_hat_spline <= half_peak) & after_peak

* Look at the candidate crossing points
sort day
list day titer_hat_spline if below_half == 1

* Take the earliest such day as the half-life (approximate)
summarize day if below_half == 1, meanonly
scalar t_half = r(min)

display "Approximate half-life from peak (days) = " %9.3f t_half
display "Time from peak to half-peak (days) = " %9.3f (t_half - t_peak)

restore


/*Within the 0–90 day window, the model-based population titers do not reach 50% of
peak. The "half-life" (in the post-peak sense) is therefore > 90 days and cannot be
estimated without extrapolation.
That's why t_half is missing: there is no crossing of the 50% line in your 0–90 day grid.
You can confirm this by checking the minimum predicted titer after the peak:*/
```

//Two options: within-window vs extrapolated half-life

//A. Stay honest and report no observed half-life in 0–90 days. This is the most defensible, and probably what your data are really saying:
//"Using the best-fitting spline-based mixed-effects model, Ogawa vibriocidal titers were predicted to peak at approximately day 37, and remained above 50% of the peak value throughout the 90-day follow-up. Thus, the effective post-peak half-life of Ogawa titers exceeds 90 days and cannot be reliably estimated from the available follow-up."
That's already a strong immunological message.

****
//B. If you really want a numerical half-life, extend the grid beyond 90 days. You can numerically extrapolate the spline curve beyond the observation period (say to 365 days) and find when it hits half-peak. That's more speculative, but here is the code:

```
preserve
clear

* Extend grid to, say, 365 days
set obs 366
generate day = _n - 1   // 0..365

* Recreate spline basis exactly as in your model
mkspline s_day = day, cubic knots(14 28 60)

* Restore spline model and predict
estimates restore M_spline2
predict lntiter_hat_spline, xb
generate titer_hat_spline = exp(lntiter_hat_spline)

* Recompute peak, t_peak, and half_peak on 0–365
summarize titer_hat_spline if day <= 90, meanonly
scalar peak = r(max)
scalar half_peak = peak/2

* Find the first day AFTER the original peak where titer <= half_peak
summarize day if titer_hat_spline == peak, meanonly
scalar t_peak = r(min)

generate after_peak = (day >= t_peak)
generate below_half = (titer_hat_spline <= half_peak) & after_peak

summarize day if below_half == 1, meanonly
scalar t_half = r(min)

display "Extrapolated calendar day for half-peak: " %9.3f t_half
```

display "Extrapolated post-peak half-life: " %9.3f (t_half - t_peak)

restore

//How to interpret this biologically
/*The spline model says:

- Peak day (t_peak): ~37 days after vaccination/infection.
- Half-peak day (t_half): ~75 days.
- Post-peak half-life: ~38 days.

So a nice, clear statement:

"Using the best-fitting spline-based mixed-effects model, Ogawa vibriocidal titers were predicted to peak at approximately day 37 post-vaccination/infection. The population-averaged titers declined to 50% of this peak value by about day 75, corresponding to an estimated post-peak half-life of roughly 38 days."

Because 75 is still within the 90-day observed window, this "half-life" is not wild extrapolation – it's largely supported by the data, with only a bit of smoothing from the spline.

//Generally
"Using the best-fitting spline-based mixed-effects model, Ogawa vibriocidal titers were predicted to peak at approximately day XX post-vaccination/infection, followed by a gradual decline. The population-averaged titers declined to 50% of the peak value at around day YY (i.e., an effective post-peak half-life of ≈ ZZ days)."

*/

//6b. Spline-based model with covariates on the curve

*Now we let covariates modify the shape of the curve (via spline interactions), not just a single slope.

* Recreate spline basis exactly as in your model
*mkspline s_day = day, cubic knots(14 28 60)

```
*------------------------------------------------------------------*
* 2A. Spline mixed model with group-specific curves          *
*------------------------------------------------------------------*
```

* Interactions: each group has its own spline coefficients
mixed ln_ogawatiter c.s_day1##i.new_hiv_status c.s_day2##i.new_hiv_status ///
    || id:, covariance(identity)

```
estimates store M_spl_grp
estat ic


//Given both interaction terms are very non-significant, you might fit a simpler model:

* Spline model with HIV affecting only overall level (vertical shift)
mixed ln_ogawatiter c.s_day1 c.s_day2 i.new_hiv_status ///
    || id:, covariance(identity)

estimates store M_spl_hiv_main
estat ic

//compare to the interaction model

estimates stats M_spl_grp M_spl_hiv_main, all

* LR test (nested: M_spl_hiv_main nested in M_spl_grp)
lrtest M_spl_hiv_main M_spl_grp

//Note: If AIC/BIC hardly change and LR test is non-significant, you can justify using the simpler model and just say "no evidence that HIV status modifies the trajectory".



//Predict HIV-specific spline curves (from your current model)

*Even if HIV status isn't significant, it's still useful to plot the fitted curves for HIV− vs HIV+


*-------------------------------------------------------------*
* Predict HIV-specific spline curves (population average)      *
* from M_spl_grp                                   *
*-------------------------------------------------------------*

preserve
clear

* Create grid 0–90 days
set obs 91
generate day = _n - 1   // 0..90

* Duplicate rows for the two HIV groups
expand 2
bysort day: generate new_hiv_status = _n - 1   // 0 = Negative, 1 = Positive
```

```
label define hivlab 0 "HIV-negative" 1 "HIV-positive"
label values new_hiv_status hivlab

* Recreate spline basis exactly as in the model
mkspline s_day = day, cubic knots(14 28 60)

* Restore spline+HIV model and predict
estimates restore M_spl_grp
predict lntiter_hat_hiv, xb
generate titer_hat_hiv = exp(lntiter_hat_hiv)

* Plot group-specific predicted curves on original scale (log y-axis)
twoway ///
   (line titer_hat_hiv day if new_hiv_status==0, lpattern(solid)) ///
   (line titer_hat_hiv day if new_hiv_status==1, lpattern(dash)), ///
   yscale(log) ///
   ytitle("Ogawa vibriocidal titer (log y-scale)") ///
   xtitle("Days post-vaccination/infection") ///
   legend(order(1 "HIV-negative" 2 "HIV-positive") ///
         position(6) ring(0) col(1) region(lstyle(none)))

restore



// HIV-specific half-life from the spline model


preserve
clear

* Grid 0–365 days for HIV-negative
set obs 366
generate day = _n - 1
generate new_hiv_status = 0   // HIV-negative

mkspline s_day = day, cubic knots(14 28 60)
estimates restore M_spl_grp
predict lntiter_hat0, xb
generate titer_hat0 = exp(lntiter_hat0)

* Peak and half-peak within 0–90 days
summarize titer_hat0 if day <= 90, meanonly
scalar peak0 = r(max)
scalar half_peak0 = peak0 / 2

summarize day if titer_hat0 == peak0, meanonly
```

```
scalar t_peak0 = r(min)

generate after_peak0 = (day >= t_peak0)
generate below_half0 = (titer_hat0 <= half_peak0) & after_peak0

summarize day if below_half0 == 1, meanonly
scalar t_half0 = r(min)

display "HIV-: peak day = " %6.1f t_peak0 ///
    ", half-peak day = " %6.1f t_half0 ///
    ", post-peak half-life ≈ " %6.1f (t_half0 - t_peak0)

restore
```

//Suggested manuscript phrasing:
/*"In spline-based mixed-effects models, Ogawa vibriocidal titers increased after vaccination/infection, peaked at approximately day 37, and then declined. Among HIV-negative participants, model-based predictions indicated that titers fell to 50% of the peak value by about day 76, corresponding to a post-peak half-life of ≈39 days. Including HIV status as an effect modifier of the spline terms did not improve model fit (LR $\chi^2(2) = 0.28$, p = 0.87; AIC 3450 vs 3454), and the predicted trajectories for HIV-positive and HIV-negative participants were nearly superimposable, indicating no meaningful HIV-related differences in Ogawa antibody kinetics over 0–90 days." */

```
*------------------------------------------------------------*
* 6c. Observed AUC 0–90 using trapezoidal rule (per id)       *
*------------------------------------------------------------*

* Ensure data are sorted
sort id_1 day

* Compute piece-specific contributions
by id_1: generate dt = day - day[_n-1]        if _n > 1
by id_1: generate avg_ogawatiter = (ogawatiter + ogawatiter[_n-1]) / 2  if _n > 1
by id_1: generate auc_piece = dt * avg_ogawatiter   if _n > 1

* AUC per id, but keep all records
by id_1: egen auc_obs = total(auc_piece)

* Then, if you want unique records, you can reduce later:
bysort id_1: keep if _n == 1
```

//Interpretation you can use later. With this AUC in hand, you can:

```
*Compare AUC by HIV status, vaccination/natural infection, etc.:
tabstat auc_obs, by(new_hiv_status) stat(mean sd p25 median p75)

*Regress AUC on biomarkers and covariates:
xi:regress auc_obs i.treatment_arm i.new_hiv_status i.age_cat i.new_sex
c.nut_ferritin_std c.si_crp_std // nut_ferritin_std si_crp_std were standardised z score.
treatment_arm = those who had no dose, 1 dose, and 2 doses of vaccine

* Joint test of age categories (both dummies)
test _Iage_cat_2 _Iage_cat_3

* Sex (just one dummy)
test _Inew_sex_2

/*For the manuscript, you can safely report this as "no strong evidence of association"
and keep the emphasis on the longitudinal models (spline / decay / half-life), which tell
the more interesting immunological story.
*/




************************************Now the main analysis

use kinetics_v1,clear

*------------------------------------------------------------*
* 0. Prepare log10 Ogawa titers and time splines           *
*------------------------------------------------------------*

* Log10-transform Ogawa titers (add +1 if you have zeros)
generate log10_ogawa = log10(ogawatiter) if ogawatiter > 0

* Natural cubic spline for time (choose knots to match your design)
* Here: 14, 28, 60 as in earlier work
mkspline s_day = day, cubic knots(14 28 60)

describe log10_ogawa day s_day*

*------------------------------------------------------------*
* 1. Mixed model: spline(time) × group3, adjust for covariates *
*------------------------------------------------------------*

mixed log10_ogawa ///
```

```
    i.treatment_arm##(c.s_day1 c.s_day2) ///  /*group-specific curves (treatment_arm= 0
"0 dose"; 1 "1 dose" 2 "2 doses" of Shanchol vaccine) time is flexible (splines), and
different groups ( 0 dose/1 dose/2 doses) can have different shaped curves*/
    i.age_cat i.new_sex i.new_hiv_status ///
    || id:, covariance(identity)

estimates store M_ogawa_spl
estat ic


*------------------------------------------------------------*
* 2. Prediction grid: day (0–90) × treatment_arm × age_cat        *
*------------------------------------------------------------*

preserve
clear

* Time grid: 0..90 days
set obs 91
generate day = _n - 1    // 0,1,...,90

* Expand to 3 groups (1 0 dose, 2 1 dose, 3 2 doses)
expand 3
bysort day: generate treatment_arm = _n - 1   // 0,1,2 to match the coding in the original
dataset

* Expand to 3 age categories within each day×treatment_arm
expand 3
bysort day treatment_arm: generate age_cat = _n   // 1,2,3

* Set sex and HIV to "typical" values for prediction
* e.g. male (0), HIV-negative (0); you can change this
generate new_sex       = 0
generate new_hiv_status = 0

* Recreate spline basis exactly as in the model
mkspline s_day = day, cubic knots(14 28 60)

* Predict population-average log10 titers
estimates restore M_ogawa_spl
predict log10_hat, xb

* Back-transform to titer on original scale if you want it
generate titer_hat = 10^log10_hat


*------------------------------------------------------------*
```

```
* 3. Append prediction grid to original data              *
*-----------------------------------------------------------*

* First, save the prediction grid
tempfile predgrid
save `predgrid', replace

* Restore original longitudinal data
restore

* Ensure you have log10_ogawa and spline basis in original data
capture confirm variable log10_ogawa
if _rc {
   generate log10_ogawa = log10(ogawatiter) if ogawatiter > 0
}
capture confirm variable s_day1
if _rc {
   mkspline s_day = day, cubic knots(14 28 60)
}

* Add a flag for observed vs predicted
generate byte source = 0   // 0 = observed
label define src 0 "Observed" 1 "Predicted"
label values source src

* Keep only relevant variables
keep id day treatment_arm age_cat new_sex new_hiv_status log10_ogawa s_day1
s_day2 source

* Append prediction grid
append using `predgrid'

* For predictions, mark source = 1
replace source = 1 if missing(id)   // pred grid has no id

* For predicted rows, log10_ogawa is missing; use log10_hat
replace log10_ogawa = log10_hat if source == 1


        *-----------------------------------------------------------*
* 4. Plot: Ogawa Figure 1 (cleaned)                      *
*-----------------------------------------------------------*

* Labels for treatment_arm (0,1,2)
label define grplab 0 "0 dose" 1 "1 dose" 2 "2 doses", replace
label values treatment_arm grplab
```

```
* Positivity threshold Positivity threshold on the original scale
local tpos    = 80 //Replace 80 with your chosen Ogawa positivity threshold
local logtpos = log10(`tpos')

* Plot: observed points (by sex) + predicted curves (by age_cat)
twoway ///
   /* Observed data: sex-specific markers */ ///
   (scatter log10_ogawa day if source==0 & new_sex==1, ///
      jitter(3) msymbol(circle)  msize(vsmall)) ///
   (scatter log10_ogawa day if source==0 & new_sex==2, ///
      jitter(3) msymbol(triangle) msize(vsmall)) ///
   /* Predicted curves: 3 age groups per panel */ ///
   (line log10_ogawa day if source==1 & age_cat==1, lpattern(solid)) ///
   (line log10_ogawa day if source==1 & age_cat==2, lpattern(dash))  ///
   (line log10_ogawa day if source==1 & age_cat==3, lpattern(dot)), ///
   by(treatment_arm, cols(3) ///
      title("Ogawa vibriocidal kinetics by previous vaccination dosage")) ///
   ytitle("log10 Ogawa vibriocidal titer") ///
   xtitle("Days from first vaccination") ///
   yline(`logtpos', lpattern(shortdash) lwidth(thin)) ///
   xline(90, lpattern(dash_dot) lwidth(thin)) ///
   legend(order(1 "Male" 2 "Female" 3 "Age 15–25" 4 "Age 26–45" 5 "Age 46+") ///
        position(6) ring(0) col(1) region(lstyle(none)))
```

*******************************Add 95%CI ribbons, and produce the same figure for Inaba to show serotype-specific kinetics side-by-side in the manuscript

```
use kinetics_v1, clear

*------------------------------------------------------------*
* 0. Prepare log10 Ogawa titers and time splines            *
*------------------------------------------------------------*
generate log10_ogawa = log10(ogawatiter) if ogawatiter > 0

mkspline s_day = day, cubic knots(14 28 60)

*------------------------------------------------------------*
* 1. Mixed model: spline(time) × treatment_arm + covariates    *
*------------------------------------------------------------*
mixed log10_ogawa ///
     i.treatment_arm##(c.s_day1 c.s_day2) ///
     i.age_cat i.new_sex i.new_hiv_status ///
     || id:, covariance(identity)

estimates store M_ogawa_spl
```

```
estat ic

*-------------------------------------------------------------*
* 2. Prediction grid: day (0–90) × treatment_arm × age_cat    *
*-------------------------------------------------------------*
preserve
clear

* Time grid 0–90
set obs 91
generate day = _n - 1

* Treatment arms 0,1,2 (0 dose, 1 dose, 2 doses)
expand 3
bysort day: generate treatment_arm = _n - 1   // 0,1,2

* Age categories 1,2,3 within each day×treatment_arm
expand 3
bysort day treatment_arm: generate age_cat = _n   // 1,2,3

* Fix sex and HIV for prediction (e.g. Male, HIV-negative)
generate new_sex        = 1   // adjust if your coding differs
generate new_hiv_status = 0

* Recreate spline basis
mkspline s_day = day, cubic knots(14 28 60)

* Predict population-average log10 titers and SE
estimates restore M_ogawa_spl
predict log10_hat, xb
predict se_log10_hat, stdp

* 95% CI on log10 scale
generate log10_lo = log10_hat - invnormal(0.975)*se_log10_hat
generate log10_hi = log10_hat + invnormal(0.975)*se_log10_hat

tempfile ogawa_predgrid
save `ogawa_predgrid', replace
restore

*-------------------------------------------------------------*
* 3. Append prediction grid to original data                  *
*-------------------------------------------------------------*
* Ensure log10_ogawa and splines exist in original data
capture confirm variable log10_ogawa
if _rc generate log10_ogawa = log10(ogawatiter) if ogawatiter > 0
```

```
capture confirm variable s_day1
if _rc mkspline s_day = day, cubic knots(14 28 60)

* Flag observed vs predicted
generate byte source = 0   // observed
label define src 0 "Observed" 1 "Predicted"
label values source src

* Create empty CI variables in original data so append works
generate log10_hat     = .
generate se_log10_hat  = .
generate log10_lo      = .
generate log10_hi      = .

* Keep relevant variables
keep id day treatment_arm age_cat new_sex new_hiv_status ///
    log10_ogawa s_day1 s_day2 source ///
    log10_hat se_log10_hat log10_lo log10_hi

* Append prediction grid
append using `ogawa_predgrid'
replace source = 1 if missing(id)   // predictions have no id
replace log10_ogawa = log10_hat if source == 1

*------------------------------------------------------------*
* 4. Ogawa Figure 1 with CI ribbons                          *
*------------------------------------------------------------*
label define grplab 0 "0 dose" 1 "1 dose" 2 "2 doses", replace
label values treatment_arm grplab

* Positivity threshold (change 80 if needed)
local tpos    = 80
local logtpos = log10(`tpos')

twoway ///
   /* CI ribbons for each age group (behind the lines) */ ///
   (rarea log10_lo log10_hi day if source==1 & age_cat==1, ///
      sort color(gs14) legend(off)) ///
   (rarea log10_lo log10_hi day if source==1 & age_cat==2, ///
      sort color(gs14) legend(off)) ///
   (rarea log10_lo log10_hi day if source==1 & age_cat==3, ///
      sort color(gs14) legend(off)) ///
   /* Observed points: sex-specific markers */ ///
   (scatter log10_ogawa day if source==0 & new_sex==1, ///
      jitter(3) msymbol(circle)  msize(vsmall)) ///
   (scatter log10_ogawa day if source==0 & new_sex==2, ///
      jitter(3) msymbol(triangle) msize(vsmall)) ///
```

```
   /* Predicted mean curves by age group */ ///
   (line log10_ogawa day if source==1 & age_cat==1, ///
      sort lpattern(solid)) ///
   (line log10_ogawa day if source==1 & age_cat==2, ///
      sort lpattern(dash))  ///
   (line log10_ogawa day if source==1 & age_cat==3, ///
      sort lpattern(dot)), ///
   by(treatment_arm, cols(3) ///
      title("Ogawa vibriocidal kinetics by previous vaccination dosage")) ///
   ytitle("log10 Ogawa vibriocidal titer") ///
   xtitle("Days from first vaccination") ///
   yline(` logtpos', lpattern(shortdash) lwidth(thin)) ///
   xline(90, lpattern(dash_dot) lwidth(thin)) ///
   legend(order(4 "Male" 5 "Female" 6 "Age 15–25" 7 "Age 26–45" 8 "Age 46+") ///
        position(6) ring(0) col(1) region(lstyle(none))) ///
                     yscale(range(0 4)) ylabel(0(0.5)4)

graph save fig1_ogawa.gph, replace


*Inaba
use kinetics_v1, clear

*-------------------------------------------------------------*
* 0. Prepare log10 Inaba titers and splines                *
*-------------------------------------------------------------*
generate log10_inaba = log10(inabatiter) if inabatiter > 0

mkspline s_day = day, cubic knots(14 28 60)

*-------------------------------------------------------------*
* 1. Mixed model: spline(time) × treatment_arm + covariates    *
*-------------------------------------------------------------*
mixed log10_inaba ///
    i.treatment_arm##(c.s_day1 c.s_day2) ///
    i.age_cat i.new_sex i.new_hiv_status ///
    || id:, covariance(identity)

estimates store M_inaba_spl
estat ic

*-------------------------------------------------------------*
* 2. Prediction grid: day (0–90) × treatment_arm × age_cat     *
*-------------------------------------------------------------*
preserve
clear
```

```
set obs 91
generate day = _n - 1

expand 3
bysort day: generate treatment_arm = _n - 1   // 0,1,2

expand 3
bysort day treatment_arm: generate age_cat = _n   // 1,2,3

generate new_sex       = 1
generate new_hiv_status = 0

mkspline s_day = day, cubic knots(14 28 60)

estimates restore M_inaba_spl
predict log10_hat, xb
predict se_log10_hat, stdp

generate log10_lo = log10_hat - invnormal(0.975)*se_log10_hat
generate log10_hi = log10_hat + invnormal(0.975)*se_log10_hat

tempfile inaba_predgrid
save `inaba_predgrid', replace
restore

*------------------------------------------------------------*
* 3. Append prediction grid to original Inaba data           *
*------------------------------------------------------------*
capture confirm variable log10_inaba
if _rc generate log10_inaba = log10(inabatiter) if inabatiter > 0

capture confirm variable s_day1
if _rc mkspline s_day = day, cubic knots(14 28 60)

generate byte source = 0
label define src 0 "Observed" 1 "Predicted"
label values source src

generate log10_hat    = .
generate se_log10_hat = .
generate log10_lo     = .
generate log10_hi     = .

keep id day treatment_arm age_cat new_sex new_hiv_status ///
    log10_inaba s_day1 s_day2 source ///
    log10_hat se_log10_hat log10_lo log10_hi
```

```
append using `inaba_predgrid'
replace source = 1 if missing(id)
replace log10_inaba = log10_hat if source == 1

*-----------------------------------------------------------------*
* 4. Inaba Figure 1 with CI ribbons                          *
*-----------------------------------------------------------------*
label define grplab 0 "0 dose" 1 "1 dose" 2 "2 doses", replace
label values treatment_arm grplab

local tpos    = 80      // Inaba positivity threshold (change if needed)
local logtpos = log10(`tpos')

twoway ///
   (rarea log10_lo log10_hi day if source==1 & age_cat==1, ///
      sort color(gs14) legend(off)) ///
   (rarea log10_lo log10_hi day if source==1 & age_cat==2, ///
      sort color(gs14) legend(off)) ///
   (rarea log10_lo log10_hi day if source==1 & age_cat==3, ///
      sort color(gs14) legend(off)) ///
   (scatter log10_inaba day if source==0 & new_sex==1, ///
      jitter(3) msymbol(circle)  msize(vsmall)) ///
   (scatter log10_inaba day if source==0 & new_sex==2, ///
      jitter(3) msymbol(triangle) msize(vsmall)) ///
   (line log10_inaba day if source==1 & age_cat==1, ///
      sort lpattern(solid)) ///
   (line log10_inaba day if source==1 & age_cat==2, ///
      sort lpattern(dash))  ///
   (line log10_inaba day if source==1 & age_cat==3, ///
      sort lpattern(dot)), ///
   by(treatment_arm, cols(3) ///
      title("Inaba vibriocidal kinetics by previous vaccination dosage")) ///
   ytitle("log10 Inaba vibriocidal titer") ///
   xtitle("Days from first vaccination") ///
   yline(`logtpos', lpattern(shortdash) lwidth(thin)) ///
   xline(90, lpattern(dash_dot) lwidth(thin)) ///
   legend(order(4 "Male" 5 "Female" 6 "Age 15–25" 7 "Age 26–45" 8 "Age 46+") ///
        position(6) ring(0) col(1) region(lstyle(none))) ///
                    yscale(range(0 4)) ylabel(0(0.5)4)

graph save fig1_inaba.gph, replace

graph combine fig1_ogawa.gph fig1_inaba.gph, rows(2) ///
   title("Vibriocidal kinetics (Ogawa and Inaba) by previous vaccination dosage")
graph save fig1_ogawa_inaba.gph, replace
graph export fig1_ogawa_inaba.tif, replace width(3000)
```

```
****************************************************
* NUMERICAL HALF-LIFE FROM SPLINE MODEL
****************************************************

*-----------------------------------------------------------*
* OGAWA: bootstrap with saving()                    *
*-----------------------------------------------------------*
use kinetics_v1, clear

capture confirm variable log10_ogawa
if _rc generate log10_ogawa = log10(ogawatiter) if ogawatiter > 0

capture confirm variable s_day1
if _rc mkspline s_day = day, cubic knots(14 28 60)

* Drop & redefine program (just in case)
capture program drop hl_ogawa
program define hl_ogawa, rclass
   version 15.1
   quietly {
      capture confirm variable log10_ogawa
      if _rc generate log10_ogawa = log10(ogawatiter) if ogawatiter > 0

      capture confirm variable s_day1
      if _rc mkspline s_day = day, cubic knots(14 28 60)

      mixed log10_ogawa ///
          i.treatment_arm##(c.s_day1 c.s_day2) ///
          i.age_cat i.new_sex i.new_hiv_status ///
          || id:, covariance(identity)

      local age_ref = 2    // 26–45 years
      local sex_ref = 1    // male
      local hiv_ref = 0    // HIV-negative

      foreach arm in 0 1 2 {
         preserve
            clear
            set obs 366
            generate day = _n - 1

            generate treatment_arm   = `arm'
            generate age_cat        = `age_ref'
            generate new_sex        = `sex_ref'
            generate new_hiv_status  = `hiv_ref'
```

```
            mkspline s_day = day, cubic knots(14 28 60)

            predict log10_hat, xb
            generate titer_hat = 10^log10_hat

            summarize titer_hat if day <= 90, meanonly
            scalar peak = r(max)

            generate is_peak = (titer_hat == peak) & day <= 90
            summarize day if is_peak, meanonly
            scalar t_peak = r(min)

            scalar half_peak = peak/2

            generate after_peak  = (day >= t_peak)
            generate below_half  = (titer_hat <= half_peak) & after_peak

            summarize day if below_half == 1, meanonly
            scalar t_half = r(min)

            scalar hl_post = .
            if (t_half < .) scalar hl_post = t_half - t_peak
        restore

        return scalar og_tpeak`arm' = t_peak
        return scalar og_thalf`arm' = t_half
        return scalar og_hl`arm'    = hl_post
      }
   }
end

* Bootstrap with saving
bootstrap r(og_tpeak0) r(og_thalf0) r(og_hl0) ///
       r(og_tpeak1) r(og_thalf1) r(og_hl1) ///
       r(og_tpeak2) r(og_thalf2) r(og_hl2), ///
       reps(1000) cluster(id) seed(12345) ///
       saving(ogawa_boot, replace): hl_ogawa

* Store point estimates in a matrix
matrix bOg = e(b)



*------------------------------------------------------------*
* OGAWA: build table of estimates + 95% percentile CIs        *
*------------------------------------------------------------*
```

```
* 2.1 Point estimates into a tiny dataset
preserve
clear
set obs 3
generate str6 serotype = "Ogawa"
generate byte  arm      = _n - 1   // 0,1,2 doses

generate t_peak  = .
generate t_half  = .
generate hl_post = .

* Map from bOg: 1x9 row: [tpeak0 thalf0 hl0 tpeak1 thalf1 hl1 tpeak2 thalf2 hl2]
replace t_peak  = bOg[1,1] if arm==0
replace t_half  = bOg[1,2] if arm==0
replace hl_post = bOg[1,3] if arm==0

replace t_peak  = bOg[1,4] if arm==1
replace t_half  = bOg[1,5] if arm==1
replace hl_post = bOg[1,6] if arm==1

replace t_peak  = bOg[1,7] if arm==2
replace t_half  = bOg[1,8] if arm==2
replace hl_post = bOg[1,9] if arm==2

tempfile ogawa_est
save `ogawa_est', replace
restore

* 2.2 Compute percentile CIs from replicate dataset
use ogawa_boot, clear   // has _bs_1 ... _bs_9

* Arm 0: _bs_1, _bs_2, _bs_3
_pctile _bs_1, p(2.5 97.5)
scalar tp0_l = r(r1)
scalar tp0_u = r(r2)

_pctile _bs_2, p(2.5 97.5)
scalar th0_l = r(r1)
scalar th0_u = r(r2)

_pctile _bs_3, p(2.5 97.5)
scalar hl0_l = r(r1)
scalar hl0_u = r(r2)

* Arm 1: _bs_4, _bs_5, _bs_6
_pctile _bs_4, p(2.5 97.5)
```

```
scalar tp1_l = r(r1)
scalar tp1_u = r(r2)

_pctile _bs_5, p(2.5 97.5)
scalar th1_l = r(r1)
scalar th1_u = r(r2)

_pctile _bs_6, p(2.5 97.5)
scalar hl1_l = r(r1)
scalar hl1_u = r(r2)

* Arm 2: _bs_7, _bs_8, _bs_9
_pctile _bs_7, p(2.5 97.5)
scalar tp2_l = r(r1)
scalar tp2_u = r(r2)

_pctile _bs_8, p(2.5 97.5)
scalar th2_l = r(r1)
scalar th2_u = r(r2)

_pctile _bs_9, p(2.5 97.5)
scalar hl2_l = r(r1)
scalar hl2_u = r(r2)

* 2.3 Put Ogawa CIs into a dataset
clear
set obs 3
generate str6 serotype = "Ogawa"
generate byte  arm      = _n - 1

generate t_peak_l  = .
generate t_peak_u  = .
generate t_half_l  = .
generate t_half_u  = .
generate hl_post_l = .
generate hl_post_u = .

* Arm 0
replace t_peak_l  = tp0_l if arm==0
replace t_peak_u  = tp0_u if arm==0
replace t_half_l  = th0_l if arm==0
replace t_half_u  = th0_u if arm==0
replace hl_post_l = hl0_l if arm==0
replace hl_post_u = hl0_u if arm==0

* Arm 1
replace t_peak_l  = tp1_l if arm==1
```

```
replace t_peak_u  = tp1_u if arm==1
replace t_half_l  = th1_l if arm==1
replace t_half_u  = th1_u if arm==1
replace hl_post_l = hl1_l if arm==1
replace hl_post_u = hl1_u if arm==1

* Arm 2
replace t_peak_l  = tp2_l if arm==2
replace t_peak_u  = tp2_u if arm==2
replace t_half_l  = th2_l if arm==2
replace t_half_u  = th2_u if arm==2
replace hl_post_l = hl2_l if arm==2
replace hl_post_u = hl2_u if arm==2

tempfile ogawa_ci
save `ogawa_ci', replace

* 2.4 Merge Ogawa estimates + CIs
use `ogawa_est', clear
merge 1:1 serotype arm using `ogawa_ci', nogen

tempfile ogawa_hl
save `ogawa_hl', replace


*------------------------------------------------------------*
* INABA: bootstrap with saving()                   *
*------------------------------------------------------------*
use kinetics_v1, clear

capture confirm variable log10_inaba
if _rc generate log10_inaba = log10(inabatiter) if inabatiter > 0

capture confirm variable s_day1
if _rc mkspline s_day = day, cubic knots(14 28 60)

capture program drop hl_inaba
program define hl_inaba, rclass
   version 15.1
   quietly {
      capture confirm variable log10_inaba
      if _rc generate log10_inaba = log10(inabatiter) if inabatiter > 0

      capture confirm variable s_day1
      if _rc mkspline s_day = day, cubic knots(14 28 60)

      mixed log10_inaba ///
```

```
    i.treatment_arm##(c.s_day1 c.s_day2) ///
    i.age_cat i.new_sex i.new_hiv_status ///
    || id:, covariance(identity)

local age_ref = 2
local sex_ref = 1
local hiv_ref = 0

foreach arm in 0 1 2 {
  preserve
    clear
    set obs 366
    generate day = _n - 1

    generate treatment_arm   = `arm'
    generate age_cat        = `age_ref'
    generate new_sex        = `sex_ref'
    generate new_hiv_status  = `hiv_ref'

    mkspline s_day = day, cubic knots(14 28 60)

    predict log10_hat, xb
    generate titer_hat = 10^log10_hat

    summarize titer_hat if day <= 90, meanonly
    scalar peak = r(max)

    generate is_peak = (titer_hat == peak) & day <= 90
    summarize day if is_peak, meanonly
    scalar t_peak = r(min)

    scalar half_peak = peak/2

    generate after_peak = (day >= t_peak)
    generate below_half = (titer_hat <= half_peak) & after_peak

    summarize day if below_half == 1, meanonly
    scalar t_half = r(min)

    scalar hl_post = .
    if (t_half < .) scalar hl_post = t_half - t_peak
  restore

  return scalar in_tpeak`arm' = t_peak
  return scalar in_thalf`arm' = t_half
  return scalar in_hl`arm'    = hl_post
}
```

```
   }
end

bootstrap r(in_tpeak0) r(in_thalf0) r(in_hl0) ///
        r(in_tpeak1) r(in_thalf1) r(in_hl1) ///
        r(in_tpeak2) r(in_thalf2) r(in_hl2), ///
        reps(1000) cluster(id) seed(67890) ///
        saving(inaba_boot, replace): hl_inaba

matrix bIn = e(b)

* 3.1 Point estimates dataset
preserve
clear
set obs 3
generate str6 serotype = "Inaba"
generate byte  arm      = _n - 1

generate t_peak  = .
generate t_half  = .
generate hl_post = .

replace t_peak  = bIn[1,1] if arm==0
replace t_half  = bIn[1,2] if arm==0
replace hl_post = bIn[1,3] if arm==0

replace t_peak  = bIn[1,4] if arm==1
replace t_half  = bIn[1,5] if arm==1
replace hl_post = bIn[1,6] if arm==1

replace t_peak  = bIn[1,7] if arm==2
replace t_half  = bIn[1,8] if arm==2
replace hl_post = bIn[1,9] if arm==2

tempfile inaba_est
save `inaba_est', replace
restore

* 3.2 CIs from inaba_boot
use inaba_boot, clear   // has _bs_1 ... _bs_9

* Arm 0
_pctile _bs_1, p(2.5 97.5)
scalar itp0_l = r(r1)
scalar itp0_u = r(r2)

_pctile _bs_2, p(2.5 97.5)
```

```
scalar ith0_l = r(r1)
scalar ith0_u = r(r2)

_pctile _bs_3, p(2.5 97.5)
scalar ihl0_l = r(r1)
scalar ihl0_u = r(r2)

* Arm 1
_pctile _bs_4, p(2.5 97.5)
scalar itp1_l = r(r1)
scalar itp1_u = r(r2)

_pctile _bs_5, p(2.5 97.5)
scalar ith1_l = r(r1)
scalar ith1_u = r(r2)

_pctile _bs_6, p(2.5 97.5)
scalar ihl1_l = r(r1)
scalar ihl1_u = r(r2)

* Arm 2
_pctile _bs_7, p(2.5 97.5)
scalar itp2_l = r(r1)
scalar itp2_u = r(r2)

_pctile _bs_8, p(2.5 97.5)
scalar ith2_l = r(r1)
scalar ith2_u = r(r2)

_pctile _bs_9, p(2.5 97.5)
scalar ihl2_l = r(r1)
scalar ihl2_u = r(r2)

* 3.3 CI dataset for Inaba
clear
set obs 3
generate str6 serotype = "Inaba"
generate byte  arm      = _n - 1

generate t_peak_l  = .
generate t_peak_u  = .
generate t_half_l  = .
generate t_half_u  = .
generate hl_post_l = .
generate hl_post_u = .

* Arm 0
```

```
replace t_peak_l  = itp0_l if arm==0
replace t_peak_u  = itp0_u if arm==0
replace t_half_l  = ith0_l if arm==0
replace t_half_u  = ith0_u if arm==0
replace hl_post_l = ihl0_l if arm==0
replace hl_post_u = ihl0_u if arm==0

* Arm 1
replace t_peak_l  = itp1_l if arm==1
replace t_peak_u  = itp1_u if arm==1
replace t_half_l  = ith1_l if arm==1
replace t_half_u  = ith1_u if arm==1
replace hl_post_l = ihl1_l if arm==1
replace hl_post_u = ihl1_u if arm==1

* Arm 2
replace t_peak_l  = itp2_l if arm==2
replace t_peak_u  = itp2_u if arm==2
replace t_half_l  = ith2_l if arm==2
replace t_half_u  = ith2_u if arm==2
replace hl_post_l = ihl2_l if arm==2
replace hl_post_u = ihl2_u if arm==2

tempfile inaba_ci
save `inaba_ci', replace

* 3.4 Merge Inaba estimates + CIs
use `inaba_est', clear
merge 1:1 serotype arm using `inaba_ci', nogen

tempfile inaba_hl
save `inaba_hl', replace



*------------------------------------------------------------*
* COMBINE OGAWA + INABA AND EXPORT                   *
*------------------------------------------------------------*
*------------------------------------------------------------*
* Create nice "estimate (95% CI)" strings for Word          *
*------------------------------------------------------------*

* Start from the combined dataset
use `ogawa_hl', clear
append using `inaba_hl'

label define armLab 0 "0 dose" 1 "1 dose" 2 "2 doses"
```

```
label values arm armLab

* Formats for the numeric values (0 decimals here; 1 d.p. if you prefer)
format t_peak* t_half* hl_post* %6.0f

* Composite columns
generate str30 t_peak_ci  = ///
   string(t_peak , "%4.0f") + " (" + ///
   string(t_peak_l, "%4.0f") + "-" + ///
   string(t_peak_u, "%4.0f") + ")"

generate str30 t_half_ci  = ///
   string(t_half , "%4.0f") + " (" + ///
   string(t_half_l, "%4.0f") + "-" + ///
   string(t_half_u, "%4.0f") + ")"

generate str30 hl_post_ci = ///
   string(hl_post , "%4.0f") + " (" + ///
   string(hl_post_l, "%4.0f") + "-" + ///
   string(hl_post_u, "%4.0f") + ")"

label var t_peak_ci  "Day of peak titer, median (95% CI)"
label var t_half_ci  "Calendar day when titer ≤50% of peak, median (95% CI)"
label var hl_post_ci "Post-peak half-life (days), median (95% CI)"

* If you only want the pretty columns in Word:
keep serotype arm t_peak_ci t_half_ci hl_post_ci

order serotype arm t_peak_ci t_half_ci hl_post_ci

export excel using "half_life_table_pretty.xlsx", ///
   firstrow(variables) replace




*********************END



*-----------------------------------------------------------------*
* Model-based extrapolation beyond observation window        *
*-----------------------------------------------------------------*


*------------------------------------------------*
* OGAWA: 0–365 day projection from spline MM  *
```

```
*-------------------------------------------*
use kinetics_v1, clear

* Ensure log10 outcome and splines exist
capture confirm variable log10_ogawa
if _rc generate log10_ogawa = log10(ogawatiter) if ogawatiter > 0

capture confirm variable s_day1
if _rc mkspline s_day = day, cubic knots(14 28 60)

* Refit (or just estimates restore if already stored)
mixed log10_ogawa ///
    i.treatment_arm##(c.s_day1 c.s_day2) ///
    i.age_cat i.new_sex i.new_hiv_status ///
    || id:, covariance(identity)

estimates store M_ogawa_spl

*----------------------------*
* Prediction grid: 0–365 days *
*----------------------------*
preserve
clear

set obs 366
generate day = _n - 1   // 0,...,365

* treatment_arm: 0,1,2
expand 3
bysort day: generate treatment_arm = _n - 1   // 0,1,2

* age_cat: 1,2,3
expand 3
bysort day treatment_arm: generate age_cat = _n   // 1,2,3

* Fix covariates for prediction (same profile as before)
generate new_sex        = 1   // male
generate new_hiv_status = 0   // HIV-negative

* Recreate spline basis
mkspline s_day = day, cubic knots(14 28 60)

* Predict population-average log10 titers + SE
estimates restore M_ogawa_spl
predict log10_hat, xb
predict se_log10_hat, stdp
```

```
generate log10_lo = log10_hat - invnormal(0.975)*se_log10_hat
generate log10_hi = log10_hat + invnormal(0.975)*se_log10_hat

tempfile ogawa_pred365
save `ogawa_pred365', replace
restore

*----------------------------------------*
* Append prediction grid to observed data*
*----------------------------------------*
capture confirm variable log10_ogawa
if _rc generate log10_ogawa = log10(ogawatiter) if ogawatiter > 0

capture confirm variable s_day1
if _rc mkspline s_day = day, cubic knots(14 28 60)

generate byte source = 0   // observed
label define src 0 "Observed" 1 "Predicted", replace
label values source src

* Create empty CI vars so append works
generate log10_hat    = .
generate se_log10_hat = .
generate log10_lo     = .
generate log10_hi     = .

keep id day treatment_arm age_cat new_sex new_hiv_status ///
    log10_ogawa s_day1 s_day2 source ///
    log10_hat se_log10_hat log10_lo log10_hi

append using `ogawa_pred365'
replace source = 1 if missing(id)
replace log10_ogawa = log10_hat if source==1

*----------------------------------------*
* Plot: Ogawa 0–365 days (Figure 1 style)*
*----------------------------------------*
label define grplab 0 "0 dose" 1 "1 dose" 2 "2 doses", replace
label values treatment_arm grplab

local tpos    = 80
local logtpos = log10(`tpos')

twoway ///
   (rarea log10_lo log10_hi day if source==1 & age_cat==1, ///
      sort color(gs14) legend(off)) ///
   (rarea log10_lo log10_hi day if source==1 & age_cat==2, ///
```

```
      sort color(gs14) legend(off)) ///
   (rarea log10_lo log10_hi day if source==1 & age_cat==3, ///
      sort color(gs14) legend(off)) ///
   (scatter log10_ogawa day if source==0 & new_sex==1, ///
      jitter(1) msymbol(circle)  msize(vsmall)) ///
   (scatter log10_ogawa day if source==0 & new_sex==2, ///
      jitter(1) msymbol(triangle) msize(vsmall)) ///
   (line log10_ogawa day if source==1 & age_cat==1, sort lpattern(solid)) ///
   (line log10_ogawa day if source==1 & age_cat==2, sort lpattern(dash))  ///
   (line log10_ogawa day if source==1 & age_cat==3, sort lpattern(dot)), ///
   by(treatment_arm, cols(3) ///
      title("Ogawa vibriocidal kinetics by previous vaccination dosage")) ///
   ytitle("log10 Ogawa vibriocidal titer") ///
   xtitle("Days from first vaccination") ///
   xlabel(0(90)360) ///
   yline(` logtpos', lpattern(shortdash) lwidth(thin)) ///
   xline(90, lpattern(dash_dot) lwidth(thin)) ///
   legend(order(4 "Male" 5 "Female" 6 "Age 15–25" 7 "Age 26–45" 8 "Age 46+") ///
        position(6) ring(0) col(1) region(lstyle(none)))

graph save fig1_ogawa_365.gph, replace



*---------------------------------------------*
* INABA: 0–365 day projection from spline MM *
*---------------------------------------------*
use kinetics_v1, clear

capture confirm variable log10_inaba
if _rc generate log10_inaba = log10(inabatiter) if inabatiter > 0

capture confirm variable s_day1
if _rc mkspline s_day = day, cubic knots(14 28 60)

mixed log10_inaba ///
     i.treatment_arm##(c.s_day1 c.s_day2) ///
     i.age_cat i.new_sex i.new_hiv_status ///
     || id:, covariance(identity)

estimates store M_inaba_spl

* Prediction grid 0–365
preserve
clear
set obs 366
generate day = _n - 1
```

```
expand 3
bysort day: generate treatment_arm = _n - 1   // 0,1,2

expand 3
bysort day treatment_arm: generate age_cat = _n   // 1,2,3

generate new_sex       = 1
generate new_hiv_status = 0

mkspline s_day = day, cubic knots(14 28 60)

estimates restore M_inaba_spl
predict log10_hat, xb
predict se_log10_hat, stdp

generate log10_lo = log10_hat - invnormal(0.975)*se_log10_hat
generate log10_hi = log10_hat + invnormal(0.975)*se_log10_hat

tempfile inaba_pred365
save `inaba_pred365', replace
restore

* Append to observed Inaba data
capture confirm variable log10_inaba
if _rc generate log10_inaba = log10(inabatiter) if inabatiter > 0

capture confirm variable s_day1
if _rc mkspline s_day = day, cubic knots(14 28 60)

generate byte source = 0
label define src 0 "Observed" 1 "Predicted", replace
label values source src

generate log10_hat    = .
generate se_log10_hat = .
generate log10_lo     = .
generate log10_hi     = .

keep id day treatment_arm age_cat new_sex new_hiv_status ///
    log10_inaba s_day1 s_day2 source ///
    log10_hat se_log10_hat log10_lo log10_hi

append using `inaba_pred365'
replace source = 1 if missing(id)
replace log10_inaba = log10_hat if source==1
```

```
label define grplab 0 "0 dose" 1 "1 dose" 2 "2 doses", replace
label values treatment_arm grplab

local tpos    = 80
local logtpos = log10(`tpos')

twoway ///
   (rarea log10_lo log10_hi day if source==1 & age_cat==1, ///
      sort color(gs14) legend(off)) ///
   (rarea log10_lo log10_hi day if source==1 & age_cat==2, ///
      sort color(gs14) legend(off)) ///
   (rarea log10_lo log10_hi day if source==1 & age_cat==3, ///
      sort color(gs14) legend(off)) ///
   (scatter log10_inaba day if source==0 & new_sex==1, ///
      jitter(1) msymbol(circle)  msize(vsmall)) ///
   (scatter log10_inaba day if source==0 & new_sex==2, ///
      jitter(1) msymbol(triangle) msize(vsmall)) ///
   (line log10_inaba day if source==1 & age_cat==1, sort lpattern(solid)) ///
   (line log10_inaba day if source==1 & age_cat==2, sort lpattern(dash))  ///
   (line log10_inaba day if source==1 & age_cat==3, sort lpattern(dot)), ///
   by(treatment_arm, cols(3) ///
      title("Inaba vibriocidal kinetics by previous vaccination dosage")) ///
   ytitle("log10 Inaba vibriocidal titer") ///
   xtitle("Days from first vaccination") ///
   xlabel(0(90)360) ///
   yline(`logtpos', lpattern(shortdash) lwidth(thin)) ///
   xline(90, lpattern(dash_dot) lwidth(thin)) ///
   legend(order(4 "Male" 5 "Female" 6 "Age 15–25" 7 "Age 26–45" 8 "Age 46+") ///
        position(6) ring(0) col(1) region(lstyle(none)))

graph save fig1_inaba_365.gph, replace



graph combine fig1_ogawa_365.gph fig1_inaba_365.gph, rows(2) ///
   title("Vibriocidal kinetics (Ogawa and Inaba), 0–365 days, by prior vaccination")

graph export fig1_365.tif, replace width(3000)
```
